# Dupilumab Facial Redness (DFR) Cleared With Oral Itraconazole

**DOI:** 10.7759/cureus.70780

**Published:** 2024-10-03

**Authors:** Drashti Devani, Disha Chakraborty, Abhishek De

**Affiliations:** 1 Dermatology, Community Health Center (CHC) Kuvadava, Rajkot, IND; 2 Dermatology, Sacramento Veterans Affairs (VA) Medical Center, Rancho Cordova, USA; 3 Dermatology, Calcutta National Medical College and Hospital, Kolkata, IND

**Keywords:** adverse effects, atopic dermatitis, dupilumab, facial redness, patient management

## Abstract

Dupilumab is the first US FDA-approved biologic for moderate to severe atopic dermatitis (AD) in adults and children of age more than six months. It is a fully monoclonal antibody that inhibits interleukin (IL)-4 and IL-13 signal transmission. The initial product monograph mentioned major side effects like hypersensitivity reactions and eye problems like conjunctivitis, dry eye, and keratitis. Persistent facial redness with dupilumab administration has been reported in the past, and itraconazole proved to be effective for its treatment. We report a case of adult AD, on treatment with dupilumab, experiencing dupilumab facial redness (DFR), giving a positive response to itraconazole. DFR is a recognized complication of dupilumab therapy for AD. Clinicians should maintain a high index of suspicion for DFR, especially in patients presenting with new-onset facial symptoms during dupilumab treatment. Itraconazole can be considered a standard therapy for DFR, given its efficacy and tolerability profile in this context.

## Introduction

Dupilumab, a fully monoclonal antibody targeting interleukin (IL)-4 receptor alpha, is the first US FDA-approved biologic for moderate to severe atopic dermatitis (AD) in adults and children over six months. Dupilumab has been recently approved by the Drugs Controller General of India and is now available in India for selected patients [1]. Though dupilumab is found to be very safe for long-term use in AD, several reports and trials highlighted ocular side effects like conjunctivitis as the major side effects. The other side effects of dupilumab include injection site reactions, eyelid redness, swelling and headache, nasopharyngitis, keratitis, and herpetic infections. Alopecia areata, pneumonia, and eosinophilia are rare side effects of the drug. However, after a few years of use, the phenomenon of persistent facial redness with dupilumab administration, first reported in April 2018, gained recognition in recent literature [2]. Despite its underreporting, this erythema commonly known as dupilumab facial redness (DFR) has been addressed in therapeutic trials, with itraconazole emerging as an effective treatment option. We report a case of adult AD, on treatment with dupilumab, experiencing DFR, giving a positive response to itraconazole.

## Case presentation

**Figure 1:**
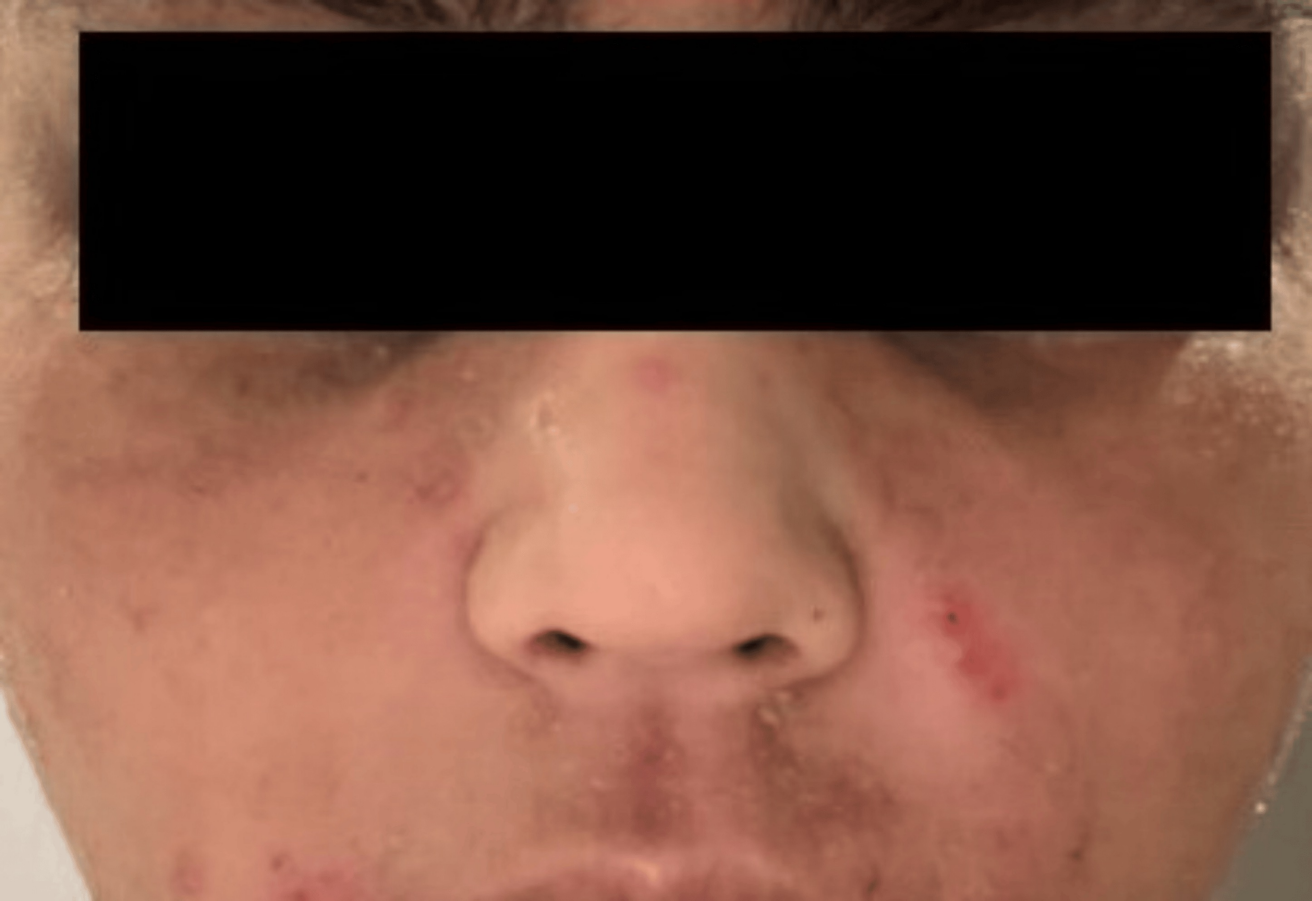
Patient with atopic dermatitis before starting dupilumab

A female patient in her 20s presented to OPD with severe AD. She had been suffering from the disease since the age of two years and had previously received various topical and oral agents including methotrexate and cyclosporine without satisfactory improvement. Upon examination, the patient exhibited erythema and scaling across the face, with a predominance over the nasolabial folds and forehead. She reported experiencing severe itching. At the presentation, she had 84% body surface area (BSA) involvement with an Investigator's Static Global Assessment (ISGA) score of 4 and an Eczema Area and Severity Index (EASI) score of 58. After basic investigations, she was started on Inj. dupilumab on a standard dosage schedule, i.e., a loading dose of 600 mg (two 300 mg injections in different injection sites), followed by 300 mg, once every 15 days (Figure 1).

**Figure 2:**
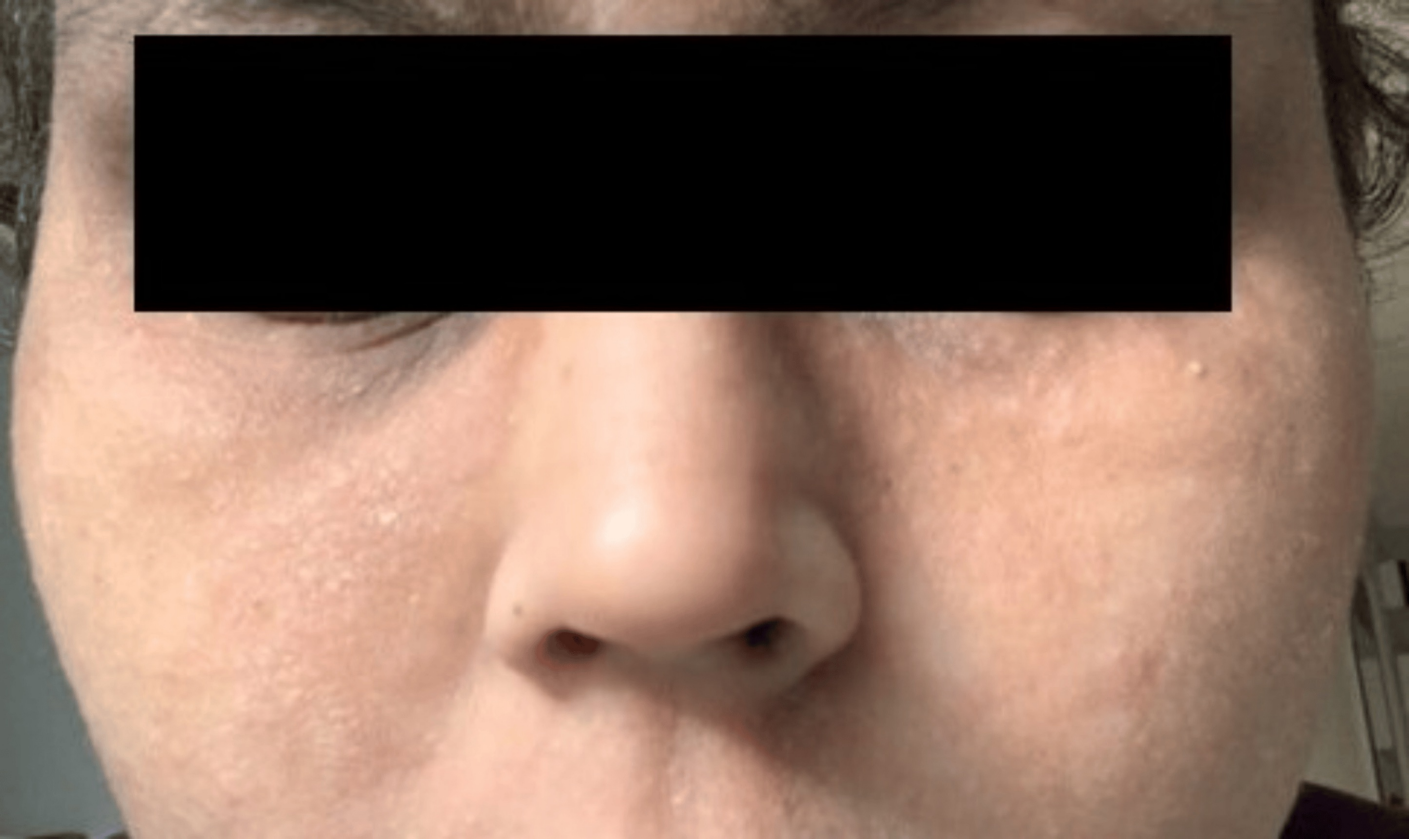
Patient with atopic dermatitis after eight weeks of dupilumab

Upon initiating dupilumab therapy, she experienced significant relief from AD symptoms. The symptoms started improving after three weeks of treatment, with a reduction in scaling and itching. Upon examination, a marked reduction in erythema and scaling was observed compared to her previous visit, with nearly clear skin noted. The erythema over the nasolabial folds had almost completely resolved. By the end of the second month of treatment, the patient had nearly clear skin in most parts of the body. At the end of eight weeks, her ISGA score was 1, her BSA involvement was 11%, and her EASI score was 8 (Figure 2). 

**Figure 3:**
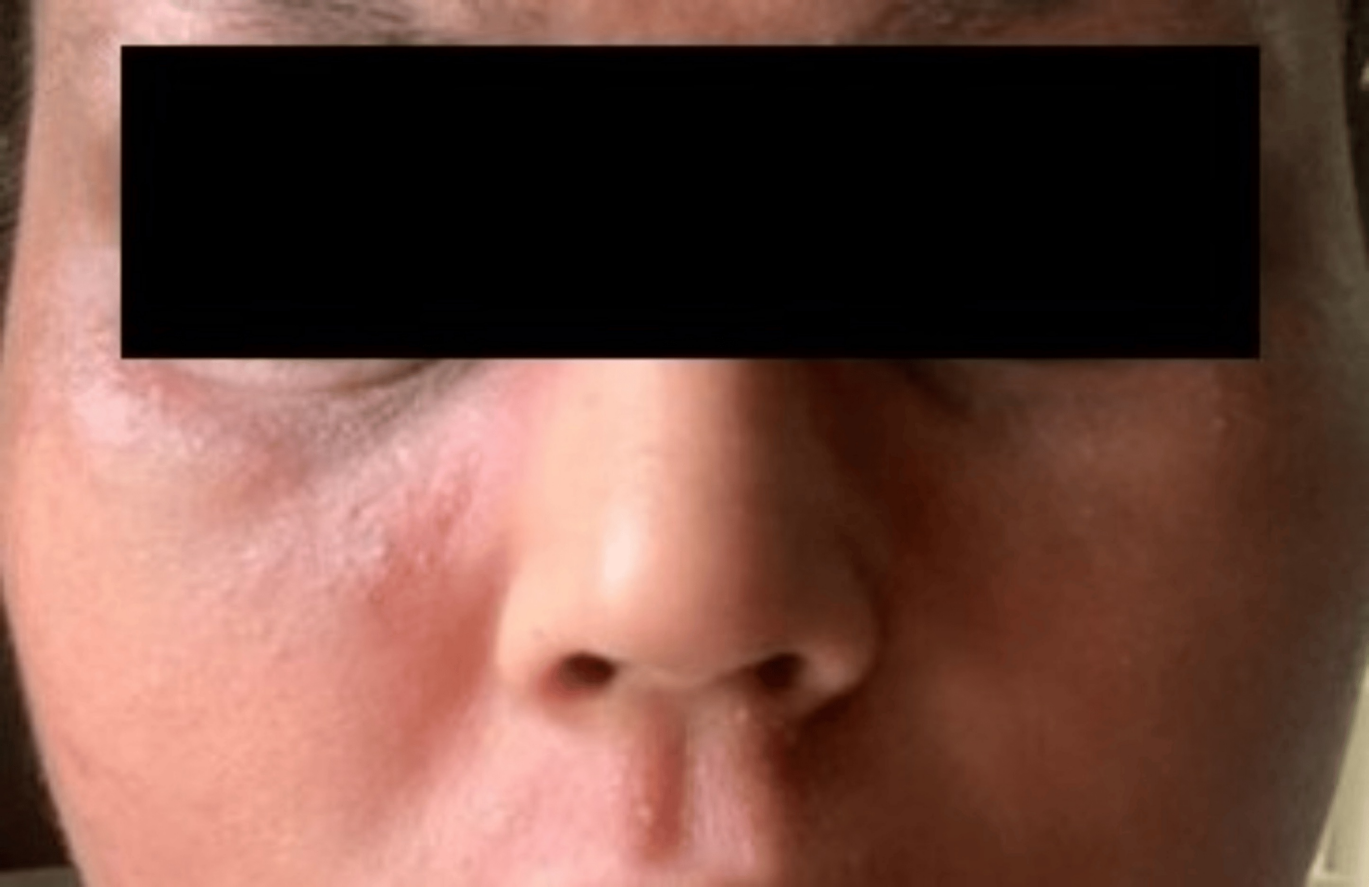
Dupilumab facial redness following three months of dupilumab

However, in the third month of treatment, she developed an erythematous rash over the face and neck, diagnosed as DFR. The new-onset facial redness was three weeks after her last dupilumab dose. She did not give any history of use of cosmetics or any other known allergens. We conducted a patch test with the Indian Standard Series, and no relevant and significant positive reaction was noted. No history of use of photosensitive drugs or products could be elicited (Figure 3). 

**Figure 4:**
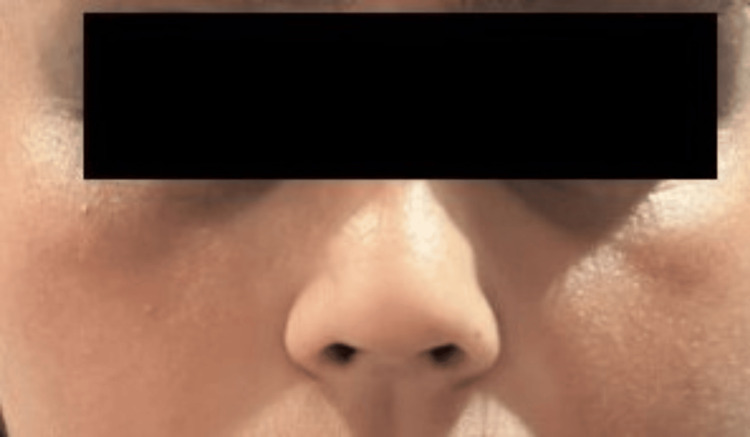
Redness cleared after six weeks of oral itraconazole

She was diagnosed with DFR and we initiated treatment with itraconazole 100 mg BD. We noted a notable clearance of the facial rash by the sixth week of therapy (Figure 4). 

We considered the differentials for this case as allergic contact dermatitis, rosacea, topical steroid-damaged faces, periorificial dermatitis (POD), demodicosis, topical steroid withdrawal, *Malassezia* dermatitis, and seborrheic dermatitis. Temporarily withholding dupilumab helped confirm the diagnosis, as her condition improved after discontinuing dupilumab. We also conducted a patch test with the Indian Standard Series, and no relevant and significant positive reaction was noted. No history of use of photosensitive drugs or products could be elicited. We initiated treatment with itraconazole 100 mg BD for six weeks. We added moisturizers and sunscreen as a part of supportive management. The erythema remained stable without exacerbation for two weeks after initiating oral itraconazole. Starting from the third week, the erythema began to diminish. We continued itraconazole for an additional three weeks as a maintenance dose, as the erythema gradually diminished, with clear skin observed at the end of six weeks.

We noted a notable clearance of the facial rash by the sixth week of therapy. We continued her dupilumab treatment as per schedule, and her skin condition stayed clear in other parts of the body.

## Discussion

DFR is a recognized side effect of dupilumab, occurring in an estimated 4-44% of recipients [3]. The duration of the onset of DFR varies. The mean has been found to be 65.4 days. However, some authors have found it to start as late as 8.5 months after the initiation of dupilumab therapy. It typically manifests as erythematous plaques with mild scaling and edema over the face and neck and may be associated with periocular dermatitis. The mechanism of DFR is still not known completely. Four theories have been proposed to explain DFR. DFR may represent a hypersensitivity reaction to dupilumab, site-specific treatment failure, seborrheic dermatitis-like reaction to facial *Malassezia* species, paradoxical flaring of allergic contact dermatitis, or a combination of these [3]. Differential diagnoses for DFR include allergic contact dermatitis, rosacea, topical steroid-damaged faces, and others. Temporarily withholding dupilumab can help confirm the diagnosis, as DFR tends to improve after discontinuation [4]. Some authors suggested that dupilumab can be held off temporarily to confirm the diagnosis of DFR, which will improve after stopping the drug [5]. 

In a review of 16 studies, DFR was noted in 101 patients. Out of them, 52% had prior AD, while 45% reported new symptoms most probably due to different underlying etiologies. Treatments included topical agents. The agents discussed here include topical corticosteroids, calcineurin inhibitors, and topical antifungal agents. All were utilized to treat AD lesions, showing favorable outcomes across various patient populations. Follow-up showed improvement in 29, clearance in four, no response in 16, and worsening in eight patients. Overall, 11% stopped dupilumab due to this issue [5]. Waldman et al.'s multi-institutional review found that DFR occurs more frequently than previously thought. Surprisingly, many patients choose to continue the medication despite this side effect. This decision is often driven by the fact that DFR is perceived as less concerning than the skin condition it is treating [6].

Various treatment options for DFR have been explored, including topical corticosteroids, topical calcineurin inhibitors, topical PDE-4 inhibitors, topical antibiotics, topical ketoconazole, and oral agents such as itraconazole. Oral itraconazole has been found effective in various reports globally. While the mechanism of action of itraconazole in DFR is unclear, its anti-inflammatory and antifungal effects are thought to play a role in its efficacy. Fluconazole has been tried for DFR, but it did not yield satisfactory improvement [7]. A single case report of DFR responding to oral ivermectin has been published [8].

## Conclusions

DFR is a recognized complication of dupilumab therapy for AD. Clinicians should be particularly alert to the possibility of DFR in patients who develop new facial symptoms while undergoing dupilumab treatment. Itraconazole can be considered a standard therapy for DFR, given its efficacy and tolerability profile in this context. We hope this case contributes to the growing understanding of DFR and aids in the management of such patients receiving dupilumab therapy for AD.
